# Computational optimization of delivery parameters to guide the development of targeted Nasal spray

**DOI:** 10.1038/s41598-023-30252-4

**Published:** 2023-03-12

**Authors:** Jinze Du, Xiecheng Shao, Jean-Marie C. Bouteiller, Angela Lu, Isaac Asante, Stan Louie, Mark S. Humayun, Gianluca Lazzi

**Affiliations:** 1 Department of Electrical Engineering, University of Southern California, Los Angeles, CA 90089 USA; 2 Department of Biomedical Engineering, University of Southern California, Los Angeles, CA 90089 USA; 3 Departments of Ophthalmology, University of Southern California, Los Angeles, CA USA; 4 Institute for Technology and Medical Systems Innovation (ITEMS), Keck School of Medicine, University of Southern California, Los Angeles, CA 90089 USA; 5 Roski Eye Institute, University of Southern California, Los Angeles, CA USA; 6 USC Allen and Charlotte Ginsburg Institute for Biomedical Therapeutics, Los Angeles, CA USA; 7 Department of Pharmacy, University of Southern California, Los Angeles, CA 90089 USA

**Keywords:** Computational biology and bioinformatics, Computational models, Epidemiology

## Abstract

Airborne transmission by droplets and aerosols is known to play a critical role in the spread of many viruses amongst which are the common flu and the more recent SARS-CoV-2 viruses. In the case of SARS-CoV-2, the nasal cavity not only constitutes an important viral entry point, but also a primary site of infection (Sungnak W. et al. Nat. Med. 26:681–687. 10.1038/s41591-020-0868-6, 2020).. Although face masks are a well-established preventive measure, development of novel and easy-to-use prophylactic measures would be highly beneficial in fighting viral spread and the subsequent emergence of variants of concern (Tao K. et al. Nat Rev Genet 22:757–773. 10.1038/s41576-021-00408-x, 2021). Our group has been working on optimizing a nasal spray delivery system that deposits particles inside the susceptible regions of the nasal cavity to act as a mechanical barrier to impede viral entry. Here, we identify computationally the delivery parameters that maximize the protection offered by this barrier. We introduce the computational approach and quantify the protection rate obtained as a function of a broad range of delivery parameters. We also introduce a modified design and demonstrate that it significantly improves deposition, thus constituting a viable approach to protect against nasal infection of airborne viruses. We then discuss our findings and the implications of this novel system on the prevention of respiratory diseases and targeted drug delivery.

## Introduction

Severe acute respiratory syndrome coronavirus 2 (SARS-CoV-2) is the pathogen responsible for the COVID-19 pandemic, which has infected more than 500 million individuals and is responsible for approximately 6 million deaths worldwide. This nascent coronavirus is primarily transmitted through aerosolized droplets, and is a dreadful reminder of the critical need to develop appropriate prevention and/or treatment methods against airborne viral infections^3–6^. In the case of SARS-CoV-2, nasal swabs obtained from symptomatic patients contained higher viral loads than in throat swabs, indicating that the nasal cavity may an important viral entry point^1,7^. In situ RNA mapping revealed the highest angiotens in converting enzyme 2 (ACE2) expression is found in the nasopharyngeal passage, further pointing to nasal orifice as the predominant site for SARS-CoV-2 infection. Furthermore, scRNA-seq datasets reveal ACE2 and its associated protease transmembrane protease serine 2 (TMPRSS2) are highly expressed in the ciliated and goblet cells of the nasal cavity^1,8^. Following infection of nasal epithelial, SARS-CoV-2 can progress to include acute respiratory distress syndrome (ARDS) and long-term sequelae^9^.

Although face masks have been established as an effective preventive measure, compliance has been moderate to poor, as well as the fact that the mask cannot be worn while eating and drinking. Therefore, the development of novel efficient and easy-to-use preemptive measures can be highly beneficial in preventing viral transmission^2,10–12^. To enhance the mucocutaneous protective barrier, we have developed a nanoparticle formulation to be delivered as a nasal spray. The formulation would act as a viral entry inhibitor, thereby protecting the susceptible epithelial cells from primary infection. Beside the intranasal delivery being used to circumvent potential drug bioavailability challenges such as poor absorption, distribution, and potential adverse events^13^, it can be explored to prevent respiratory infections in the nasopharyngeal cavity.

The nasal cavity has a complex convoluted geometry that provide efficient filtration of the inhaled air and enhanced olfaction. However, this convoluted geometry prevents the coating of the nasal mucosa with sprayed therapeutics using direct velocity from the source (i.e., using the velocity field when exiting the spray head), no matter where the intranasal source is positioned in the cavity. Thus, depending on the therapeutic aims and the properties of the drug to be delivered, different delivery strategies can be used^14^. Our therapeutic target is to enhance mucocutaneous layers found on the nasopharyngeal epithelial cells by depositing sprayed nanoparticles to enhance mucocutaneous barrier protection from infectious virions. To ensure maximum protection, a targeted delivery of the sprayed nanoparticles to maximize deposition overlapping potential deposition of inhaled virus particles. The present study describes the approach to maximize this co-deposition. We first introduce our computational approach as well as the three-dimensional nasal cavity model used; followed by the conditions and parameters relevant for our study and the different quantification metrics. The results obtained were analyze as a function of a broad range of delivery parameters. We introduced a modified design and demonstrated significant improvement for co-deposition of protective and infectious particles. Finally, we discuss the significance of our results and the applications of this novel prophylactic system for respiratory disease prevention and targeted drug delivery.

## Methodology

### 3D model of human nasal cavity

**Figure 1:**
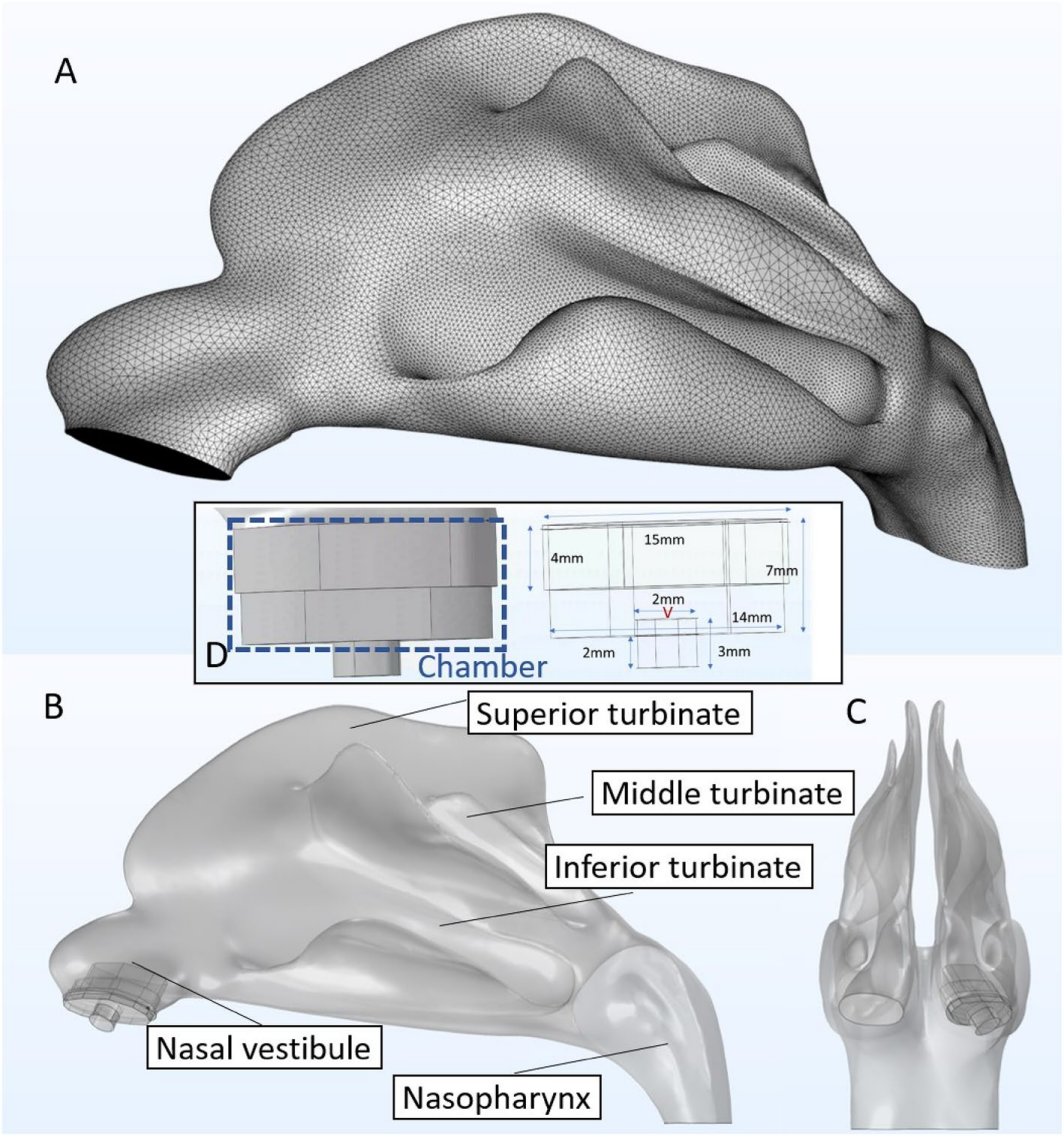
Three-dimensional nasal cavity model and spray head geometry. (**A**) Statistical average shape model of the human nasal cavity as proposed by^15^. (**B, C**) Side and front views of the nasal cavity model with the spray head inserted in the left nostril. (**D**) Dimensions of the spray head with the mixing chamber (top 7mm portion); the location at which the spray particles are released is at the center of the lower 2mm diameter head (red V).

To address the issue of nasal cavity variability between genders, individuals and ages, generation of synthetic geometries generated with the population mean overall shape variance was used. Thus, a 3D model of the nasal cavity representing the average geometry generated using a statistical shape model^15^. For this retrospective investigation, anonymized computed tomography (CT) data of 25 symptom-free subjects was used. Due to the retrospective nature of this study, the 3D CT image data were acquired using different devices with a voxel resolution of 0.37 × 0.37 × 0.4 mm or better. Then, the image data was segmented slice by slice, beginning from the frontal coronal slice (from anterior to posterior). Both sides of the nasal cavity were segmented using a flood fill tool that connected all contiguous regions. In order to further utilize this model for computational models and simulations of different nasal spray particle deposition analyses, we imported the constructed nasal cavity model into COMSOL. We meshed it into small triangular shapes with ultra-high resolution, as shown in Fig. 1. With over six million meshed triangle surfaces, we can assign these surfaces with distinct commonly observed anatomical characteristics, and we can simulate with high realism the aerodynamics of particles inhaled into the nasal cavity and their interactions with the nasal walls. More specifically, the boundary conditions governing the interactions between the internal wall of the nasal cavity and the particles were set to a “50% trap” condition, meaning that particles touching a wall, there is a 50% chance that this particle will deposit at that location^16^. If the particle does not deposit and bounces off the cavity wall, it will continue its path following the airflow; more details on this are provided in the section of the COMSOL simulations setup. Using an anatomically accurate high-resolution nasal cavity enables us to simulate the aerodynamics and tracing of viral and spray particles inhaled into the nasal cavity, thereby allowing optimization of the nasal spray design and its parameters to maximize the protection rate against COVID viral infections.

### Computational modeling of the spray: Addition of diffusion chamber

**Figure 2:**
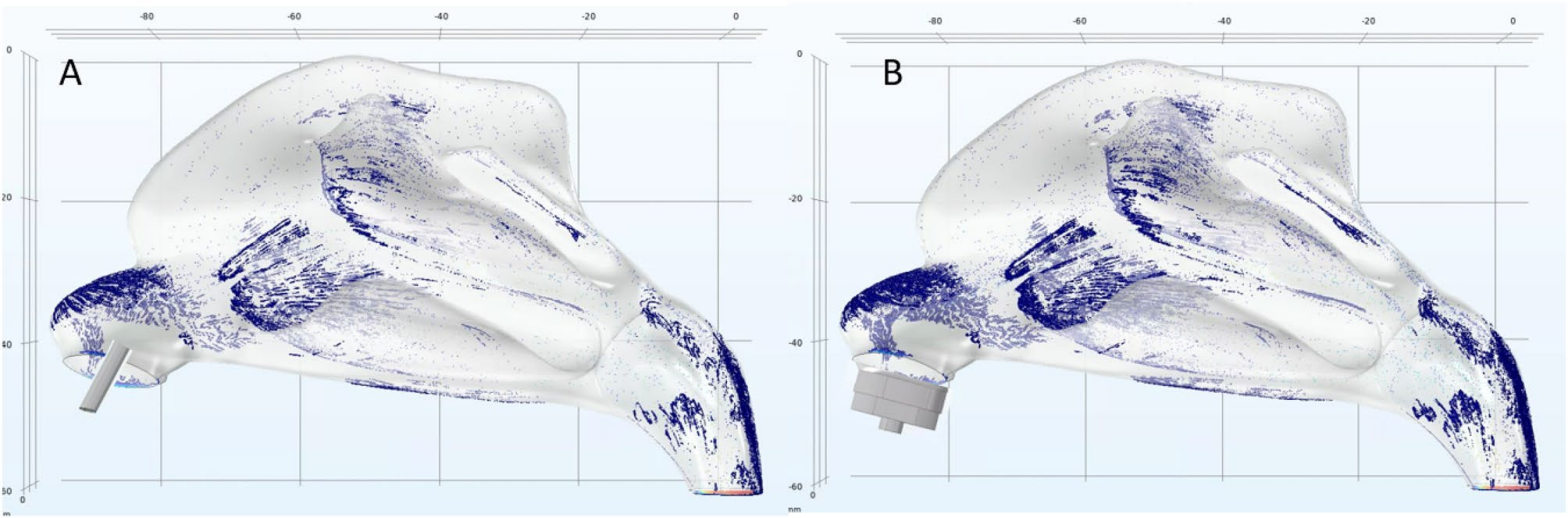
Illustration of the deposition profiles obtained with two distinct spray designs. The same parameters are used for inhalation speed (0.68m/s), cone angle (60°), spray speed (12m/s). (**A**) Deposition without chamber. (**B**) Deposition with chamber. The spray particle deposition rate of A is 16.41%, the deposition rate with diffusion chamber is about 31.09%.

Initial simulation results indicated poor deposition of sprayed particles in the posterior region of the nasal cavity, consistent with results previously reported by Inthavong^17^ (see Fig. 2). Simulation without a diffusion chamber showed that particles following the laminar flow (not their initial velocity) will travel further into the posterior region. To improve distribution in the posterior region of the nasal cavity, a diffusion chamber aimed at maximizing the probability that particles will follow the laminar flow to reach the posterior region of the cavity. The distance from the spray outlet to the chamber outlet is 7mm. The geometry of the diffusion chamber was designed to maximize the distance from the spray outlet to the exit of the diffusion chamber and ensure that most of the spray particles will lose their momentum created from the initial spray velocity when exiting the chamber. The diffusion chamber was designed to let inhaled air in through the bottom portion and have a cross-section that fits the entry of the nasal cavity in order not to affect the airflow into the nose. To ensure the velocity field inside of nasal cavity replicates normal breathing conditions, the inhalation speed was modeled 1.3 times that of baseline inhalation speed (i.e., without chamber); we use the same baseline inhalation speed of 0.68m/s for all simulations, except where otherwise stated.

### Simulation environment: COMSOL setup

With the rapid development of CPU technology, computational modeling has become a powerful tool that can guide the design and optimization of biomedical devices from wearable devices^18–21^, implantable devices^22,23^ to external usage devices such as nasal sprays. COMSOL Multiphysics is one of the most used simulation environments among different options. COMSOL Multiphysics is one of the most used simulation environments among different options. COMSOL Multiphysics was used for the simulation of inhalation and particle deposition. A velocity field of 0.68m/s^24^ using laminar flow to simulate the inhalation at a steady state. The fluid property of density and dynamic viscosity was chosen based on on-air property. A no-slip wall condition was chosen to mimic the environment inside the nasal cavity. A stationary study was chosen to simulate the steady-state airflow during inhalation. Built upon the stationary study of laminar flow was the particle tracing for the fluid flow module to simulate the particle position during laminar flow. Two different particles were used for this simulation: (1) liquid particles with a dynamic viscosity of 4.05mPa*s^25^ to mimic the liquid property of viral particles; (2) liquid particles with a dynamic viscosity of 2mPa*s to mimic the property of sprayed particles. The viscosity of the sprayed particle was obtained from lab test of the spray fluid. Drag force using Stokes law, Brownian force, and gravity force was implemented as the major force that will affect particles trajectory. For the binding mechanics of the particles, freeze wall condition was applied to both diffusion chamber and nasal cavity wall, the nasal cavity wall has 100% chance of binding with the particles whereas the diffuser wall having a 50% chance of stick or bounce condition. This condition was chosen to simulate the interactions particles-wall; these may be subsequently updated to incorporate the effect of mucus.

### Quantification of the simulation results: Deposition profile and spray protection rate

**Figure 3:**
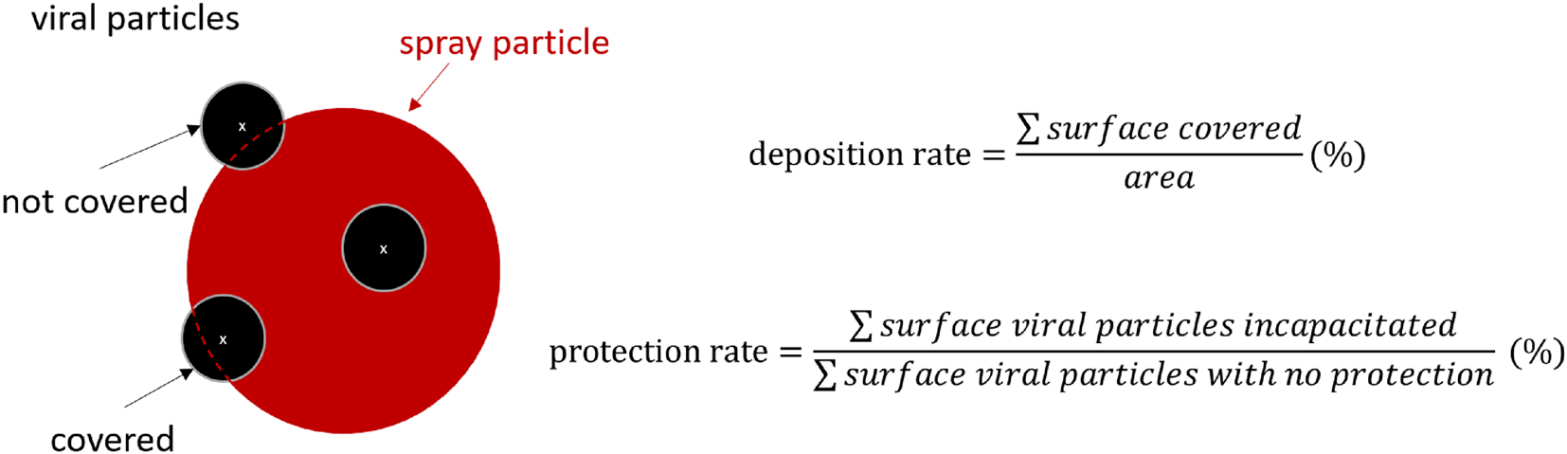
Illustration of the quantification process used to determine deposition and protection against viral particles depending on the characteristics and deposition location of spray particles (red circle) and viral particles (black circles).

To quantitatively assess the protection rate of different spray conditions with various parameters values and determine the most effective combinations, we first simulated viral particles as naturally inhaled at a speed of 0.68m/s (corresponding to 35l/min, equivalent to baseline inhalation speed^24^). The locations at which the viral particles deposit inside the nasal cavity are saved (x,y,z coordinates) in conjunction with the particles’ diameter. Various sprays with different design parameters and delivery conditions (spray velocity, cone angle, spray dose) are then simulated to obtain their distinct deposition profiles. Then a corresponding protection rate is calculated. To calculate this protection rate, deposited viral particles inside nasal cavity walls are compared against deposited spray particles using the center coordinates of the deposited particles and their diameters; this allows us to determine if a spray particle covers a viral particle. Specifically, we compare the diameter and center of each particle; if the distance between the center of the viral particle and the center of the spray particle is smaller than the diameter of the spray particle, then we determine that this spray particle fully protects against the viral particle. Otherwise, the spray particle would not protect the viral particle as only partly exposed to the nasal cavity could also cause infection. If a sprayed particle fully covers a viral particle, this particle is considered to protect against the viral particle fully. The protection rate is a generalization of these rules to all viral particles deposited in the nasal cavity, as shown in Fig. 3.

## Results

### Viral particle deposition profile

A study of influenza A viral particles emitted during flu season in a day-care facility and onboard commercial passenger flights found viral droplets sizes varied from 2.5 μm to sizes inferior to 250nm, with 64% of the load associated with particles of less than 250nm, which can remain airborne for hours^26,27^. Consistent with these results, and given that particles smaller than 5 μm in diameter are consistently generated in a large number of situations such as breathing, speaking, coughing, singing, and sneezing^28^, we simulated viral entry as the inhalation of 300,000 viral particles with diameters defined by a uniform density function, with diameters ranging from 50 nm to 5 μm. Notably, such a high number of particles exceeds reasonable exposure during one single inhalation and instead would represent a long cumulative exposure. Using such a high number of particles helps ensure that we have a large sample size of viral particles that deposit in the nasal cavity, which proves helpful for optimization. To determine the protection rate, the first step consists in evaluating the deposition of viral particles in the nasal cavity. To do so, we simulated particles mentioned before using COMSOL. Notably, our study focused on relatively small particle sizes for two main reasons. One, larger particles have been shown to drop rapidly due to their weight, thus decreasing the chance of subsequent inhalation. Virus-containing particles with diameters smaller than 5 μm are emitted in a large number of situations (breathing, speaking, coughing, singing, and sneezing^28^) and stay longer, suspended in the air, subject to environmental airflow, and are more prone to subsequent inhalation. We assume that the velocity of the viral particles as they enter the nasal cavity is 0.68m/s, equivalent to normal inhalation conditions.

### Spray particle deposition: Effect of spray particle size

**Figure 4:**
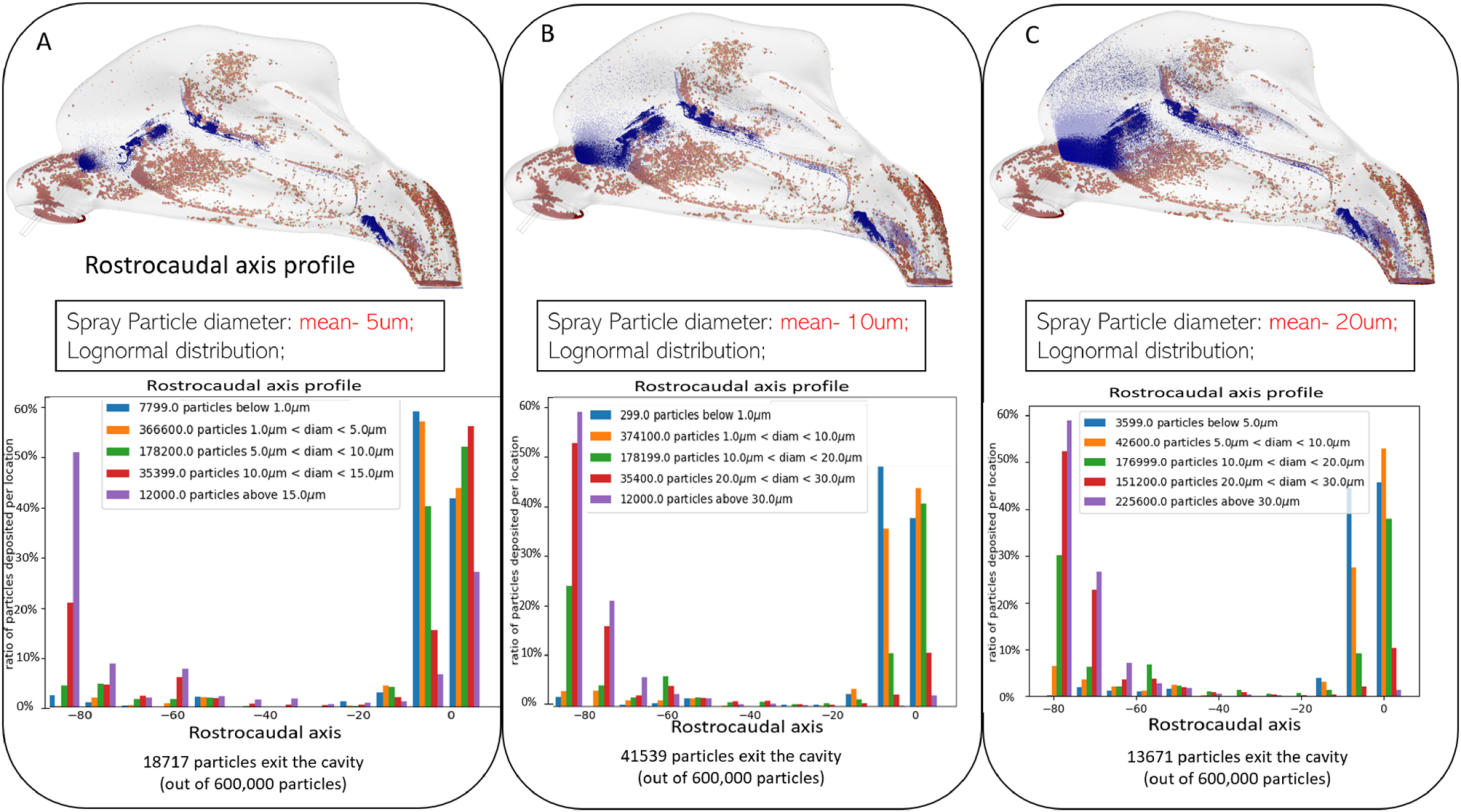
Effect of mean sprayed particle size on deposition profile. Deposition profiles obtained with a spray head inserted at 15mm inside the nasal cavity with particles following three different particle distribution functions with a mean particle diameter of 5, 10 and 20 μm. All other parameters are held the same: spray cone Angle: 60°; spray speed: 4m/s; particles number: 600,000; inhalation speed: 0.68m/s. Top: Visualization of the deposition profiles obtained. The brown particles represent the simulated viral particles deposited in the cavity, while the blue particles are deposited spray particles. Bottom: Quantification of the deposition for different particle sizes along the rostrocaudal axis. The histograms represent the ratio of particles of a certain size that land in portions of the nasal cavity binned every 10mm along the rostrocaudal (Y) axis. These results indicate that particles with a smaller diameter predominantly deposit in most posterior locations in the cavity, and even tend to exit the cavity heading toward the nasopharyngeal region. On the other hand, larger particles predominantly deposit in the anterior portion of the cavity. Taken together, these results suggest that spraying with particles that follow a more uniform distribution function (i.e., containing particles of varying sizes) results in a more uniform deposition, thereby yielding a better overall protection.

We further explored the effect of sprayed particle sizes on deposition in the nasal cavity. To do this, three configurations in which the same number of particles (600,000) was sprayed into the nasal cavity. However, the mean diameter of these particles was set to 5, 10, and 20 μm respectively, with all configurations following a log-normal distribution. All other conditions remained the same, and the results are illustrated in Fig. 4. The brown particles represent the location of viral particles while the blue particles represent the sprayed particles. The results indicate that the deposition with the smallest particles is very spotty compared to the one obtained with the 10 μm and 20 μm configurations, and the smaller particles tend to travel at the most anterior portion of the cavity heading toward the lower airway. As most commercially available nasal sprays use 50–140 μl per actuation^29^, we opted to use a total sprayed volume of 140μl as this would generate a higher number of sprayed particles deposited in the nasal cavity, thus potentially yielding a better protection rate against viral particles.

### Spray particle deposition: Effect of spraying cone angle

**Figure 5:**
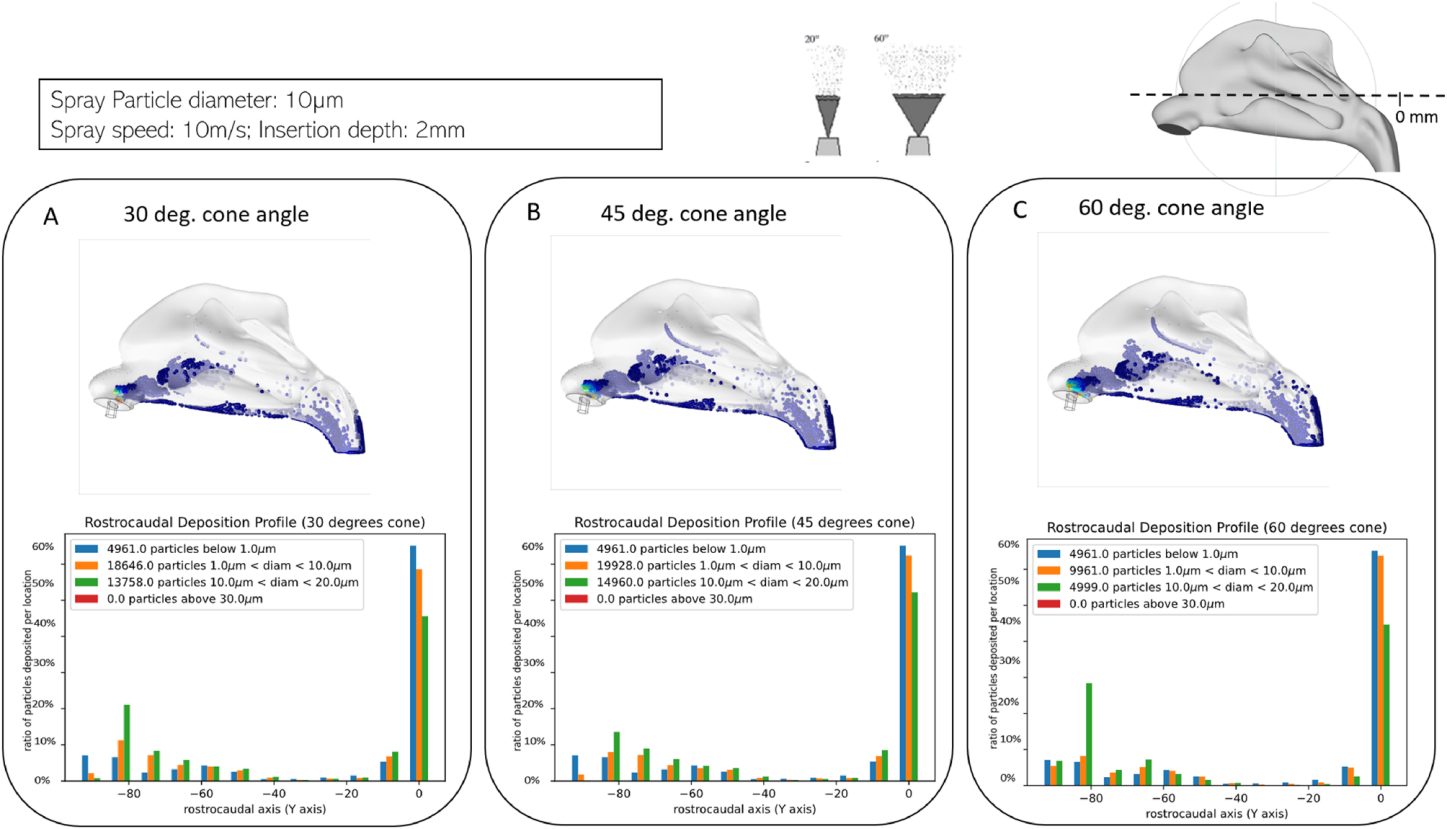
Effect of spray cone angle. Deposition profiles obtained with particles sprayed at three different angles: 30° (**A**), 45° (**B**) and 60° (**C**). All other parameters are held the same (mean particle diameter: 10 μm, a spray velocity of 10m/s, an inhalation speed of 0.68m/s and an insertion depth of 2mm). Top: Visualization of the deposition profiles obtained. The blue particles represent the spray particles deposited in the cavity. Bottom: Quantification of the deposition for the different spray angles along the rostrocaudal axis. The histograms represent the ratio of particles of a certain size that land in portions of the nasal cavity binned every 10mm along the rostrocaudal (Y) axis.

Spray cone angle has long been known to be an essential factor that affects the deposition efficacy of nasal sprays^17,30–33^. The spray cone angle describes the spray plume development, whereas the dispersion angle describes the liquid sheet fluctuation from the mean cone spray angle. The dispersion angle is an essential parameter in the LISA break-up model, as it leads to the radial droplet dispersion from the mean cone spray angle^34^; Cone angles (also referred to as ’plume angles’) of 30, 45 and 60° were studied specifically as they constitute common values in regularly used ranges^30,35^. As shown in Fig. 5, the impact of spray cone angle on the particle deposition profile varies by particle size. Variation in cone angle has the most apparent impact on particles with a diameter between 1 μm and 20 μm and shows little effect on particles larger than 20 μm. Also, observation of the deposition pattern indicates that when the cone angle is too small, e.g., 30°, the spray particles’ deposition area is smaller than the cases of 45° and 60°. To be more specific, fewer spray particles were deposited onto the inferior nasal concha area in the figure, which suggests that the 30° cone angle spray results in less protection against viral particles; this result is in complete agreement with results obtained in our parameter sweep study (“8” in section).

### Spray particle deposition: Effect of spray velocity

**Figure 6:**
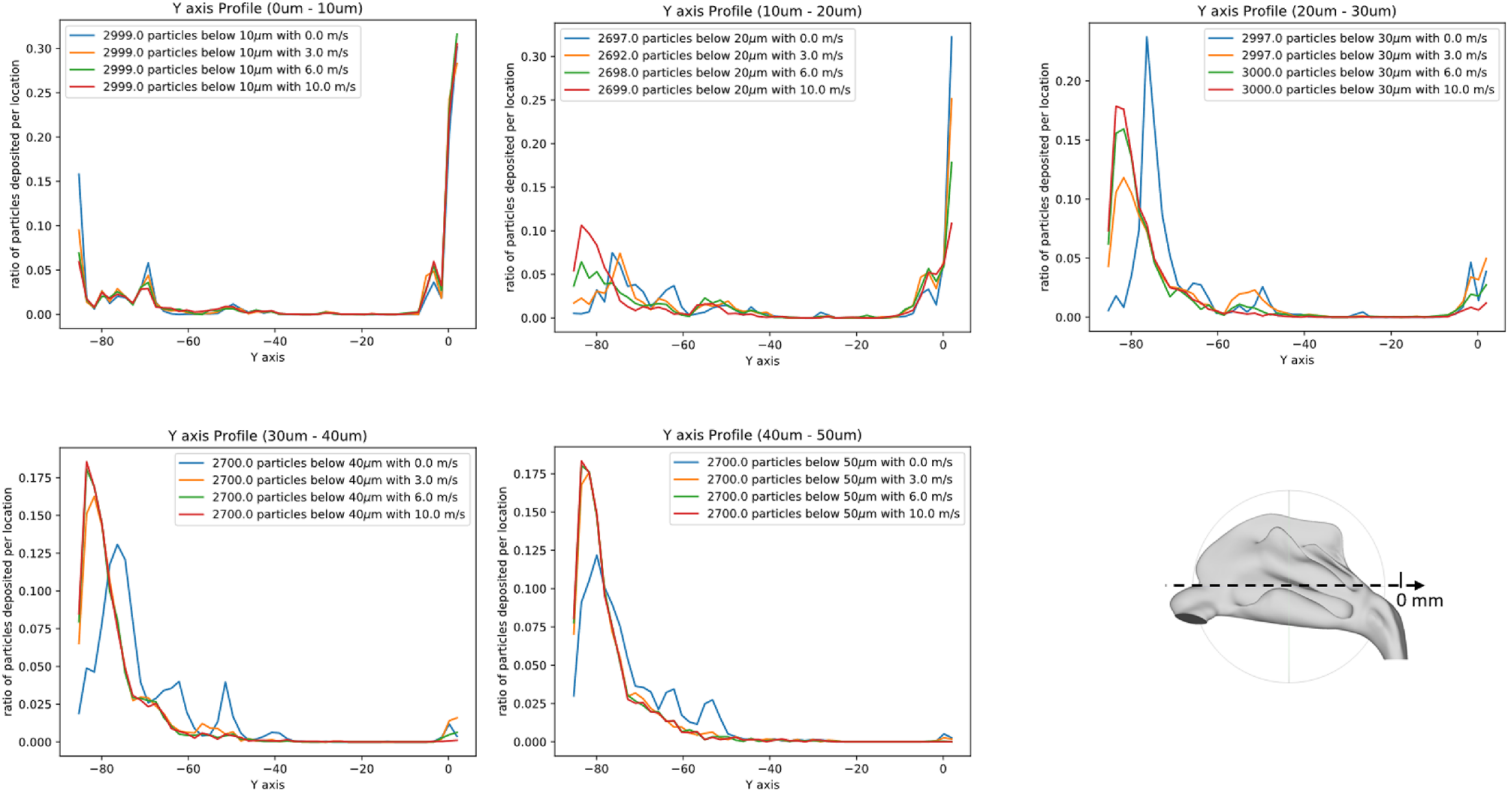
Effect of spray velocity on sprayed particle distribution. Distribution profiles of particles of varying diameters along the rostrocaudal (Y) axis. Each curve represents one simulation with different spray velocities: 0 m/s, 3 m/s, 6 m/s and 10 m/s.

Droplet aerosol velocity is an important parameter that can be used to characterize nasal spray products^36,37^. Injected particle velocities were controlled via the COMSOL simulation environment, and a range of different spray velocities was simulated from 0 m/s to 10 m/s. The results presented in Fig. 6 show the number of particles deposited along the rostrocaudal axis (denoted ’Y’ on the figure) of our nasal cavity model for different particles sizes and different velocities to determine how particles deposition profile changes with different spray velocities. Similar to the results we obtained for spray cone angle, we can observe that the effect of spray velocity on deposition profile varies widely depending on the particles considered; spray velocity appears to have very little effect on smaller particles (with a diameter < 10 μm) or larger particles ( diameter > 40 μm); instead, the strongest effect is observed on particles with a diameter in the 10–30 μm range (except for the 0 velocity, which represents a more *theoretical* case and for which inhalation alone is responsible of the airflow in the nasal cavity).

### Sprayed particles deposition: Effect of spray insertion depth

**Figure 7:**
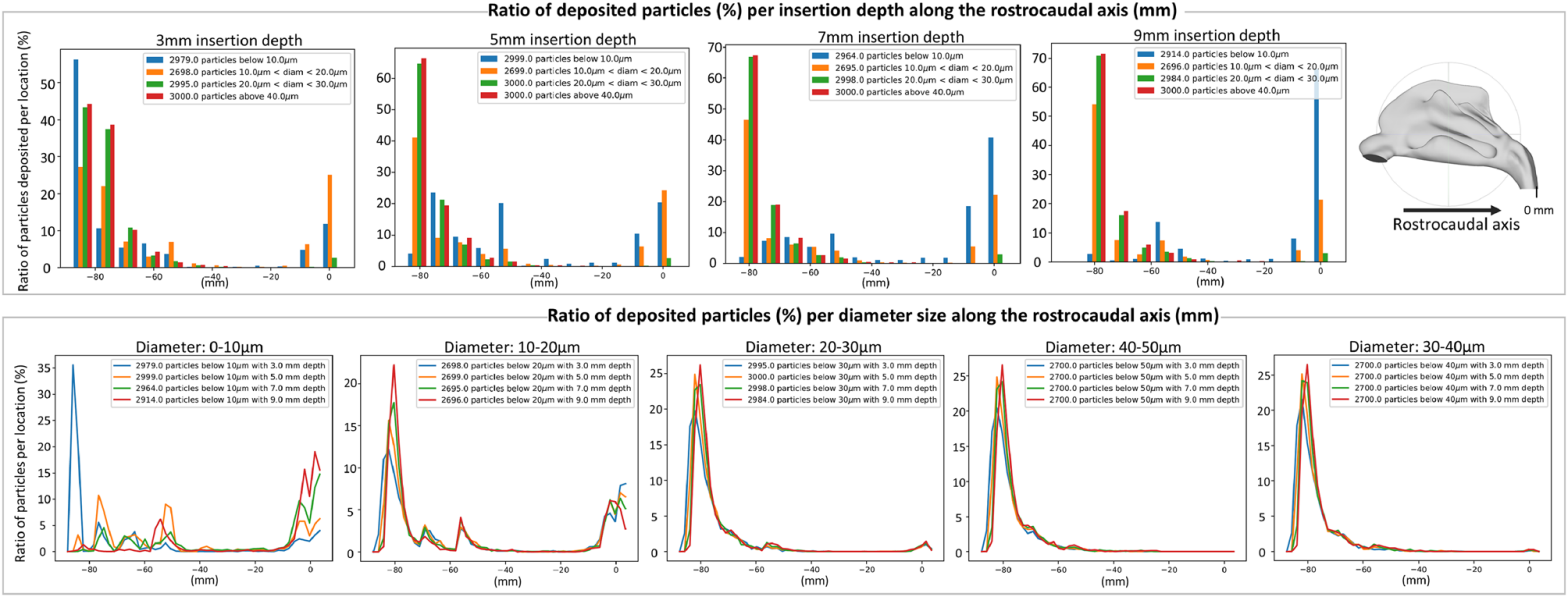
Effect of spray insertion depth on particle deposition profile for different particles sizes. Top: Profile of particles deposited is recorded for different insertion depths. Bottom: We focus on particles of different sizes and examine how insertion depth affects deposition along the rostrocaudal axis. Insertion depth appears to have little influence on the deposition profile of larger particles. On the other hand, deeper insertion results in more smaller particles depositing deeper in the nasal cavity and down the laryngopharynx region.

Topical intranasal sprays are amongst the most commonly prescribed therapeutic options for sinonasal diseases. Thus, a large number of studies are conducted in such areas. Yet, inconsistency and ambiguity in the instructions provided to the end user suggest a lack of definitive knowledge on best spray use techniques and a potential lack of understanding of the quantitative impact of different delivery parameters on the desired outcome. Insertion depth is one of these parameters that has been ’left behind’, and how deep a spray should be inserted into the nostrils is very often unclear. Some studies indicate that insertion of 10mm into the nostril improves deposition^35,38^. However, this improvement was noted to be small compared to the effect of other parameters such as insertion angle. In order to investigate how insertion depth affects our spray deposition profile, we studied the nasal spray deposition profiles with 3mm, 5mm, 7mm, and 9mm insertion depth. The particle deposition profiles were obtained with a cone angle of 45°, a spray velocity of 10m/s, and a broad range of particles diameters of up to 50 μm, which allowed us to evaluate the effect of insertion depth on the deposition of particles with very different sizes. The results shown in Fig. 7 indicate the deposition for different particle sizes along the rostrocaudal axis.

These results indicate that at low insertion depth, a more significant number of particles tends to deposit close to the nasal entry independently from their sizes. As the insertion depth increases, smaller particles become capable of traversing the nasal cavity and even exiting the cavity in the laryngopharynx region (which corresponds to a deposition at $Y=0$).

### Maximization of protection rate against SARS-CoV-2: addition of a mixing chamber and results of a comprehensive parameter sweep

Two significant factors of the design and efficacy of nasal spray, cone angle and spray velocity have long been studied on their own. Our simulation results further demonstrate that using different cone angles and spray velocities result in significantly different deposition profiles, even using physically identical spray designs. However, cone angle and spray velocity also mutually affect each other, i.e., one optimal cone angle for a specific velocity will not necessarily yield the best protection rate with another spray velocity condition. This is also true when the size of the sprayed particles is taken into consideration as large particles will more strongly follow the direction of the initial spray velocity vector, while smaller particles will more loosely follow the airflow in the nasal cavity. These observations, in conjunction with our simulation results outline that optimizing deposition of sprayed particles to mimic that of viral particles requires a more profound modification of the delivery system. The updated design would allow sprayed particles of varying diameters (shown to more uniformly deposit in the cavity) to follow the path of viral particles, i.e., the path dictated by inhalation rather than that resulting from the initial spray velocity field. Such realization resulted in the incorporation of the mixing chamber described in detail in the methods section. We now propose to thoroughly establish the efficacy of this modified delivery design while simultaneously varying cone angle and spray velocity. Notably, this design incorporates inhalation speed as an additional delivery parameter.

**Figure 8:**
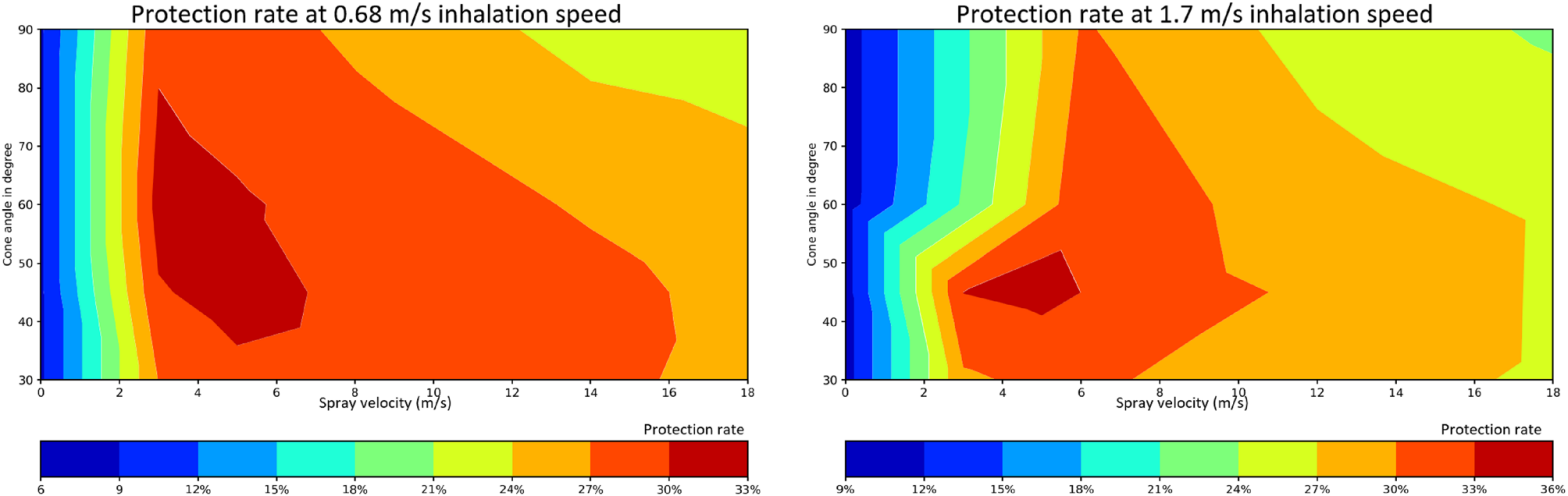
Exhaustive parameter space exploration to maximize protection rate Parameter sweep was conducted on two different scenarios with different inhalation speed: 0.68m/s and 1.7m/s. Under both circumstances, low spray velocity cannot achieve much protection against viral particles. A spray velocity of around 5m/s should yield the best protection rate when combined with 45° cone angle.

We propose to explore all possible combinations to design a nasal spray that can yield the best potential protection against viral particles. Now with the help of our powerful computational simulation platform, a parameter sweep was done and shown in Fig. 8. The parameter sweep was conducted under two different natural inhalation speed profiles: one is inhaling air with a speed of 0.68m/s, and the other is inhaling at 1.7 m/s. First, it was evident that with low spray velocity, we cannot achieve much protection against viral particles under both inhalation speed circumstances. Furthermore, the spray velocity is not the higher the better. When the spray velocity is too large, the spray particle deposition profiles might be too different from the viral scenario, and thus the protection rate suffers. Based on our simulation results, a spray velocity of around 5m/s should yield the best protection rate when combined with a proper cone angle, which is 45° in this case. A total of 36% protection rate is achieved in this scenario, which means for the total of 300000 viral particles inhaled into the nasal cavity, 36% of these viral particles will be blocked by our previously sprayed barrier particles from contacting the nasal cavity wall. The infection rate is thus reduced by 36%.

### Sprayed protective barrier versus surgical mask: Comparison of protection rate

**Figure 9:**
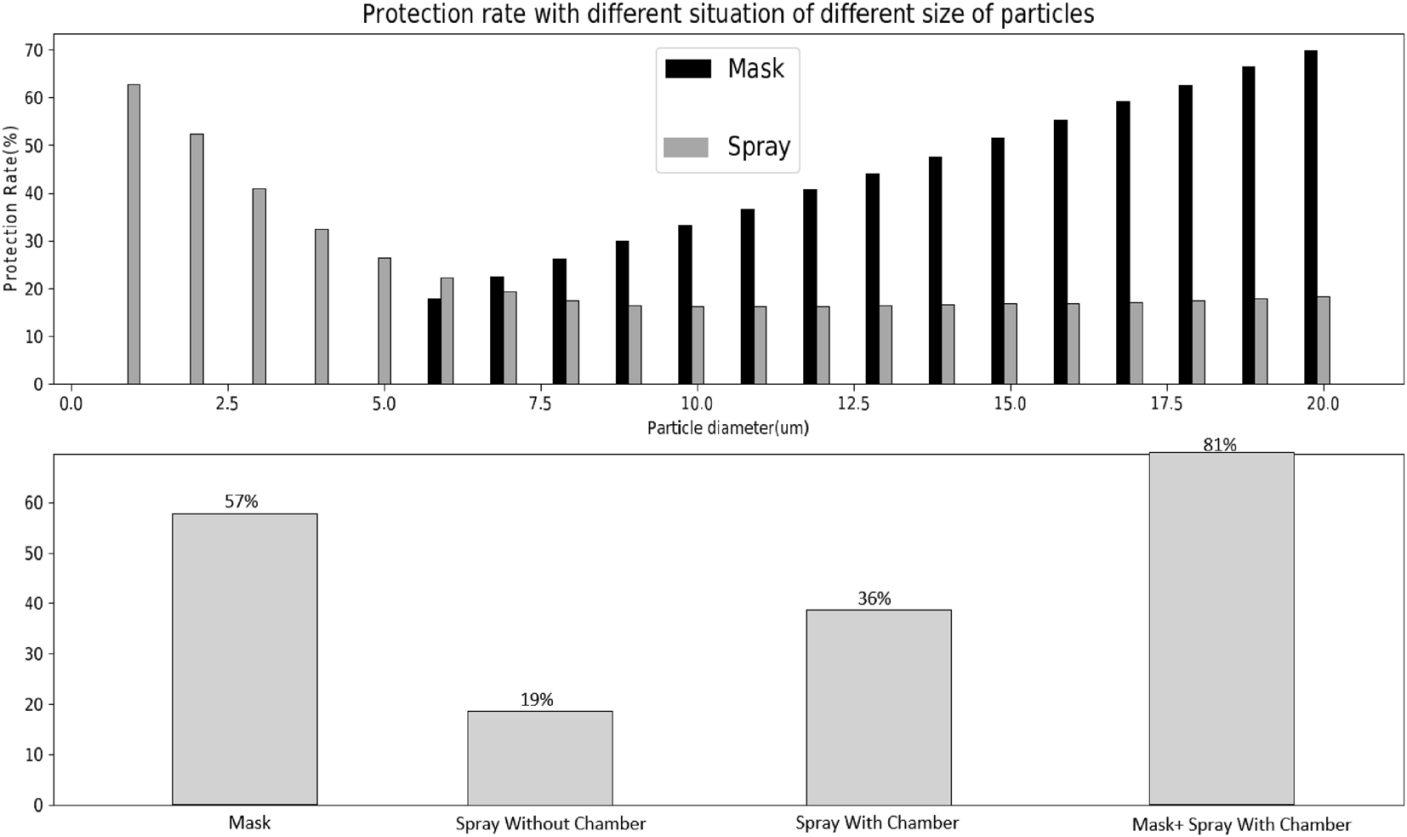
Comparison of the protection rates obtained with a surgical mask and our sprayed protective barrier. Upper graph: Protection rate against COVID-19 viral particles of different sizes with mask and spray separately. Lower graph: Total protection rate of mask, spray without chamber, spray with chamber and mask+spray with chamber. The results illustrate masks have low protection against viral particles with a diameter smaller than 5μm, however with the designed spray it can provide up to 60% protection rate against small viral particles. Thus, when mask and spray are used together highly desirable protection is achieved.

It has been well established that masks do not offer a complete protection against SARS-CoV-2 viral particles^39^. To simulate the mask protection rate, mask filtration data are obtained^10,40–42^ and modeled using the same uniformly distributed viral particle profile. The mask protection rate is then compared against our nasal spray results. In order to calculate the protection rate offered by the surgical mask, mask filtration rates of different-sized viral particles are used to simulate the particle number that can pass the mask protection. As shown in Fig. 9, our results indicate that the simulated masks are more effective at protecting against larger viral particles, but crucially lack protection for viral particles with a diameter smaller than 5 μm. On the contrary, our proposed nasal spray with diffusion chamber used with the best parameter combination identified above yields a protection rate that is much better for smaller viral particles. As illustrated in Fig. 9, masks have poor protection against viral particles with a diameter smaller than 5 μm. However, the designed spray can provide up to a 60% protection rate against small viral particles. Furthermore, once again, the protection rate demonstrated here shows the importance and effectiveness of our diffusion chamber design. Nasal spray with the same parameter without a diffusion chamber only yields a total of 19% protection rate against viral particles. However, incorporating our innovative diffusion chamber design further improved protection, resulting in a nearly two-fold increase in protection rate. Furthermore, the protection characteristics of mask and our modified design can combine and further improve the protection rate against viral particles. As outlined in the figure, Results obtained with this combination reach highly desirable protection at over 80%, which suggests that this combination could be a very effective measure to further enhance the protection against the viral particles.

## Discussion

Airborne transmission via infectious aerosolized droplets is a critical factor in the spread and transmission of SARS-CoV-2. In SARS-CoV-2, aerosolized transmission primarily infects susceptible cells found in the nasal cavity. The SARS-CoV-2 virus, in particular, has been established to have a high rate of infectivity in nasal epithelial cells due in part to their high ACE2 expression when compared to cells located deeper in the respiratory tract1. Pertinent to this observation, we investigated the use of an intranasal spray to deliver protective nanoparticles that would deposit at susceptible sites found in the nasal cavity, thereby creating a mechanical barrier atop the mucocutaneous layer capable of protecting susceptible cells. After describing the 3D nasal cavity model and the overall simulation parameters and conditions, we presented the computational approach capable of comprehensively characterize the effects of multiple delivery parameters: particle size, spray cone angle, spray velocity and insertion depth. Analysis of the results obtained while independently varying these parameters led to several observations.

Our results demonstrated that the size of sprayed particles has a critical impact on the deposition profile. In particular, the behavior of large, sprayed particles strongly depends on their inertia; they consequently tend to follow a straight path more easily, with a trajectory strongly dictated by the initial spray velocity field. Given the tortuous shape of the nasal cavity, these particles deposit on the anterior portion of the cavity, independently from cone angle and spray velocity. On the contrary, smaller particles are capable of following the tortuous airflow throughout the cavity. In general, these smaller particles can therefore reach the more posterior regions of the cavity, even reaching the laryngopharynx area for the smallest ones. These results are consistent with previous observations that larger particles tend to deposit close to the entry of the nasal cavity and its anterior portion^43^, while smaller particles are more prone to follow the airflow and land deeper in the nasal cavity, heading toward the laryngopharynx and the lower respiratory tract. Taken together, these results also indicate that if we aim to achieve a uniform deposition throughout the nasal cavity, we should use a relatively uniform particle density function that contains particles of different sizes.

We then focused on investigating the effect of *spray cone angle and initial velocity*. Although our previous results indicated that a uniform particle distribution function seemed desirable to maximize deposition, the spray velocity appears to act in a counterproductive manner as it forcibly projects the particles on the walls of the anterior portion of the cavity as a function of their inertia, rather than placing them in the airflow that would deposit them more uniformly throughout. *Maximizing the protection* against viral particles inherently entails that the protective barrier we intend on creating with the sprayed particles is created in the same anatomical regions as the viral particles, i.e., from a deposition standpoint, maximizing protection is equivalent to maximizing co-deposition. Yet the trajectory of the virus-loaded particles is predominantly dictated by the inertia of the particles and the airflow in the cavity. We therefore proposed to integrate a chamber between the spray head and the nasal cavity that would reduce the effect of initial spray velocity and allow the particles to mix with the inhaled airflow before they enter the nasal cavity. Once in the airflow, the sprayed particles can follow a path that is similar to the viral particles and hence deposit in the same anatomical locations. We verified the added benefit of this design which resulted in an improved protection rate from 19% to 36% (with all other parameters remaining equal). We then performed a systematic optimization and sensitivity analysis on this modified design for different inhalation speeds, cone angles and initial spray velocities and obtained the theoretical optimal therapeutic window for the improved design. When simulating particle deposition and calculating the rate of protection, we assumed 100% stickiness on cavity walls with deterministic laminar air flow and 50% stickiness on diffuser walls. Theoretically, the particle deposition process is subject to stochasticities. However, when we compared the results of multiple simulations, we found that the variability was practically non-existent.

In summary, this study presented and characterized a modified spray delivery system which parameters were optimized to maximize co-deposition of sprayed particles with viral particles. A large number of simulations allowed the identification of an optimal range of delivery parameters that maximizes protection; the theoretical protection obtained is approaching that of surgical masks with a single application - thereby suggesting that two (or more) applications could result in protection that is similar and possibly even better than that obtained with surgical masks. Of importance, the study presented has several limitations. One of the major limitations is that it does not take into consideration nasal mucociliary clearance which transports the mucus layer that covers the nasal epithelium towards the nasopharynx by ciliary beating. This clearance will result in a movement of the particles deposited in the cavity toward the posterior portion toward the nasopharynx^44–46^. Instead, this study focuses on optimizing the initial co-deposition phase. In addition, this study modeled discrete particles that are non-deforming, inert, with no breakup or coalescence, which results in simulations that are simpler and more efficient. This simplification may result in modeling errors that are more pronounced in the near-nozzle region of the spray but should theoretically vanish as particles enter the cavity since their concentration becomes more spread out.

The study was limited in the scope of parameters that were optimized. Although it is clear that the viscosity of the sprayed particles should be maintained in the lower range of the spectrum to help maintain a relatively low particle diameter and ensure deeper deposition^47^, this study did not fully consider the physical properties of the formulation and their consequences on spray characteristics. Notably, physical properties like formulation viscosity may also be easily adjusted to obtain the desired spray characteristics^29,48^, binding and unbinding kinetics will be dependent upon the physicochemical properties of the particles. Introducing these additional considerations and parameters would distract from the main message of the manuscript, i.e., optimizing delivery parameters to ensure a maximum protection at time zero. We therefore abstracted these considerations and used particles with a given functional characteristic in terms of stickiness and binding/unbinding properties. Of importance, considerations about the physicochemical properties of the solution sprayed will also be critical after deposition as the viscosity will affect mucosal and cilial clearance and consequently the protection over time. Additional limitations also stem from uncertainties in modeling a realistic scenario. Amongst these are the characteristics of the viral particles to which the subject is exposed. We used viral particles with a uniform distribution to take into consideration the effect of particles of varying diameters on the location at which these viral particles deposit; we also focused on relatively small viral particles ranging from 50nm to 5 μm as these are known to be exhaled in a large number of social situations and remain in the air for a longer amount of time, while bigger (and consequently heavier) particles drop more quickly to the ground. In addition, potential differences between the shape of the individual’s nasal cavity and the average shape model as well as mucosal load used may affect airflow and particles stickiness to the nasal walls. The equations governing airflow assume incompressible flow with negligible heat transfer. The velocity of the viral particles as they enter the nasal cavity is associated with baseline inhalation (i.e., with no physical activity); this velocity contains no other additional environmental component (e.g., wind). Another limitation is modeling the effects of different nebulizers. There are a variety of nebulizers on the market, such as powered nebulizers, pulsation membrane nebulizers, vibrating mesh nebulizers, etc. However, nasal nebulizers are plagued by high lung deposition and relatively low nasal delivery fractions. In order to maximize nasal deposition, each of these various nebulizers may have distinct user instructions. Some patients, for instance, may need simultaneous aspiration from the contralateral nostril and specific breathing instructions. The resulting different shear forces will have impact on the mAbs. Although previous studies showed evidence that during spray production, mAbs are frequently more sensitive to air-bubble entrainment, adsorption to solid surfaces (with possible shear synergy), contamination by particulates than to shear forces. This remains to be another limitation^49,50^. Our computational study assumed different steady inhalation speeds from one single nostril. Similarly, the initial velocity is assumed to be identical for all particles; in reality this is likely subjected to fluctuations. An exhaustive analysis of the numerous aspects mentioned above represents a tremendous task and burdensome computational load and is thus outside the scope of this paper. However strict constraints were used whenever possible to ensure the validity of the results presented. Within these constraints, we characterized the proposed modified spray delivery system with mixing chamber and its optimized delivery parameters for maximum co-deposition with viral particles.

Future studies will investigate whether incorporating a swirling spray^17,51^ to the current design could further improve co-deposition. Further investigations will also include verifying the computational results by 3D printed models. We decided not to publish the 3D printed model experiments at this time for two primary reasons, although this work is still in progress and will be presented in a forthcoming publication. The two reasons are: 1. The purpose of the work is to determine the optimal parameters for the design of a nasal spray that maximizes the virus protection rate. Many parameter combinations are simulated computationally with tiny increments in different parameter sweeps. Even carrying out all these simulations computationally was very time consuming, which makes experimental validation of all the parameter sweeping conditions even more difficult. 2. Experimental validation is still ongoing, but it is taking too long to complete, so we have decided to publish the computational work first, followed by the experimental results in a subsequent work. Further investigations will also determine how the protection evolves over time as mucosal clearance takes place and the sprayed protective barrier is displaced toward the posterior nasopharyngeal region. This will be critical in determining the kinetics and dosing parameters of the barrier. To conclude, we present a generalizable approach that has been targeted toward maximizing protection against SARS-CoV-2 viral infection, however, it can be adapted to other pathogens and their specific infection profile. It can also be applied in different exposure conditions (e.g., exposure to larger viral particles in very crowded environments) that can be readily extended to the prevention of other respiratory diseases. Using this approach to identify the optimal drug delivery parameters to ensure localized delivery, the approach presents a powerful tool for the advancement of targeted drug delivery.

## Data Availability

The datasets generated and/or analysed during the current study are not publicly available due the size being too large to upload online. But are available from the corresponding author on reasonable request.
